# Molecular understanding of sterically controlled compound release through an engineered channel protein (FhuA)

**DOI:** 10.1186/1477-3155-8-14

**Published:** 2010-06-25

**Authors:** Arcan Güven, Marco Fioroni, Bernhard Hauer, Ulrich Schwaneberg

**Affiliations:** 1 School of Engineering and Science, Jacobs University Bremen, Campus Ring 1, 28759 Bremen, Germany; 2 Lehrstuhl für Biotechnologie, RWTH Aachen University, Worringerweg 1, 52074, Aachen, Germany; 3 Institut für Technische Biochemie, Universität Stuttgart, Allmandring 31, D-70569 Stuttgart, Germany

## Abstract

**Background:**

Recently we reported a nanocontainer based reduction triggered release system through an engineered transmembrane channel (FhuA Δ1-160; Onaca *et al*., 2008). Compound fluxes within the FhuA Δ1-160 channel protein are controlled sterically through labeled lysine residues (label: 3-(2-pyridyldithio)propionic-acid-N-hydroxysuccinimide-ester). Quantifying the sterical contribution of each labeled lysine would open up an opportunity for designing compound specific drug release systems.

**Results:**

In total, 12 FhuA Δ1-160 variants were generated to gain insights on sterically controlled compound fluxes: Subset A) six FhuA Δ1-160 variants in which one of the six lysines in the interior of FhuA Δ1-160 was substituted to alanine and Subset B) six FhuA Δ1-160 variants in which only one lysine inside the barrel was not changed to alanine. Translocation efficiencies were quantified with the colorimetric TMB (3,3',5,5'-tetramethylbenzidine) detection system employing horseradish peroxidase (HRP). Investigation of the six subset A variants identified position K556A as sterically important. The K556A substitution increases TMB diffusion from 15 to 97 [nM]/s and reaches nearly the TMB diffusion value of the unlabeled FhuA Δ1-160 (102 [nM]/s). The prominent role of position K556 is confirmed by the corresponding subset B variant which contains only the K556 lysine in the interior of the barrel. Pyridyl labeling of K556 reduces TMB translocation to 16 [nM]/s reaching nearly background levels in liposomes (13 [nM]/s). A first B-factor analysis based on MD simulations confirmed that position K556 is the least fluctuating lysine among the six in the channel interior of FhuA Δ1-160 and therefore well suited for controlling compound fluxes through steric hindrance.

**Conclusions:**

A FhuA Δ1-160 based reduction triggered release system has been shown to control the compound flux by the presence of only one inner channel sterical hindrance based on 3-(2-pyridyldithio)propionic-acid labeling (amino acid position K556). As a consequence, the release kinetic can be modulated by introducing an opportune number of hindrances. The FhuA Δ1-160 channel embedded in liposomes can be advanced to a universal and compound independent release system which allows a size selective compound release through rationally re-engineered channels.

## Introduction

A channel protein that is embedded in an impermeable membrane offers the possibility to develop novel triggered drug release systems with potential applications in synthetic biology (pathway engineering), and medicine (drug release). So far only FhuA [1], OmpF [2-4], Tsx [5] and MscL [6] have been reconstituted functionally into synthetic block copolymers or lipid membranes.

FhuA is a large monomeric transmembrane protein of 714 amino acids located in the *E. coli *outer membrane folded into 22 anti-parallel β-strands and two domains [7]. By removing the "cork" domain (deletion of amino acids 5-160 [8,9]) the resulting deletion variant behaves as a large passive diffusion channel (FhuA Δ1-160) [1]. FhuA and engineered variants have a significantly wider channel than OmpF (elliptical cross section of OmpF is 7*11 Å [10] whereas FhuA is 39*46 Å [1]) allowing the translocation of even single stranded DNA [11]. Recently we reported an exclusive translocation of calcein through an engineered transmembrane FhuA Δ1-160 which had been embedded in a tri-block copolymer membrane PMOXA-PDMS-PMOXA; where PMOXA = poly(2-methyl-2-oxazoline) and PDMS = poly(dimethyl siloxane); and could be opened up through a reduction triggered system [12]. The reported calcein release kinetics were strongly modulated by the size of employed lysine-labeling reagents [12]. Twenty nine lysines are present in the FhuA Δ1-160; 19 lysines located on the protein surface, 6 are inside the channel, and 4 are at the barrel rim [12]. The 19 lysines on the FhuA surface point into the outer membrane and are after purification covered by oPOE rendering pyridyl-labeling unlikely.

An average of four lysine residues per FhuA Δ1-160 was determined to be pyridyl labeled [12]. Based on the hypothesis that the 6 lysine inside the channel might mainly be responsible for restricting compound fluxes, two subsets of FhuA Δ1-160 variants were generated. In the six subset (A) variants only one of the six lysines in channel interior was substituted by alanine and in the six subset (B) variants only one lysine remained in the channel interior whereas all other five were substituted to alanine. For the in total 12 investigated FhuA Δ1-160 variants a HRP based colorimetric TMB (3,3',5,5'-tetramethylbenzidine) detection system [13,14] was employed for quantifying the sterical hindrance of pyridyl-labeled lysines on the TMB substrate. The colorimetric HRP/TMB detection was preferred over the previously reported calcein detection system due to a higher reproducibility [1,12]. Furthermore liposomes instead of a polymeric nanocontainer system were selected for characterizing the 12 FhuA Δ1-160 variants due to more simple and rapid assay procedures [15], despite drawbacks like leakiness, stability over time [16] and undesired biomolecule adsorption on the surface [17].

However, the better kinetic results reproducibility using liposomes compared to polymersomes, where the FhuA Δ1-160 insertion can be affected by block co-polymer poly-dispersity and traces of residual chemicals, suggested us to use liposomes correcting the kinetic results by the small leakage contribution (see Table 1). To our best knowledge we report a first detailed mutational study on a transmembrane channel protein to gain, on the molecular level, first insights on the sterically controlled diffusion of TMB through the FhuA channel interior modulated by labeled lysines. Interestingly only one single lysine position is the main responsible of the TMB diffusion.

**Table 1:** Average TMB conversions in liposomes.

	FhuA Δ1-160 variant reconstituted in liposomes	**Position(s) of Lys**→**Ala Substitution(s)**	TMB conversion [nM]/s	*True averaged TMB conversion [nM]/s	**TMB conversion ratio
**Controls**	Lacking FhuA Δ1-160	-	13 ± 2	-	1

	Unlabeled FhuA Δ1-160	-	102 ± 5	89	7.9

	Fully labeled FhuA Δ1-160 starting variant	-	15 ± 4	2	1.2

**Subset A**	K167A	167	59 ± 2	46	4.5
	
	K344A	344	52 ± 1	39	4
	
	K364A	364	20 ± 3	7	1.5
	
	K537A	537	76 ± 3	63	5.9
	
	K556A	556	97 ± 4	84	7.5
	
	K586A	586	14 ± 1	1	1.1

**Subset B**	K167	344, 364, 537, 556, 586	22 ± 2	9	1.7
	
	K344	167, 364, 537, 556, 586	23 ± 1	10	1.8
	
	K364	167, 344, 537, 556, 586	30 ± 3	17	2.3
	
	K537	167, 344, 364, 556, 586	21 ± 3	8	1.6
	
	K556	167, 344, 364, 537, 586	16 ± 1	3	1.2
	
	K586	167, 344, 364, 537, 556	35 ± 2	22	2.7

Lambert Beer law was used with an extinction coefficient of 3.9 × 10^-4 ^M^-1 ^cm^-1 ^for the first TMB oxidation product. Two subsets (A & B) of FhuA Δ1-160 variants were apart from controls analyzed. FhuA Δ1-160 variants of subset A contain a single lysine to alanine substitution while subset B contain five lysine to alanine substitutions. All FhuA Δ1-160 variants are pyridyl-labeled except two controls (liposome lacking FhuA Δ1-160 and the unlabeled FhuA Δ1-160). "*": The true TMB conversion is calculated from the TMB-conversion of FhuA Δ1-160 variant subtracted by the TMB conversion of the background lacking FhuA Δ1-160; "**": TMB conversion ratio represents a ratio between TMB conversions of pyridyl-labeled FhuA Δ1-160 variants and the liposome control lacking FhuA Δ1-160.

## Results

### FhuA Δ1-160 based compound release system

**Figure 1:**
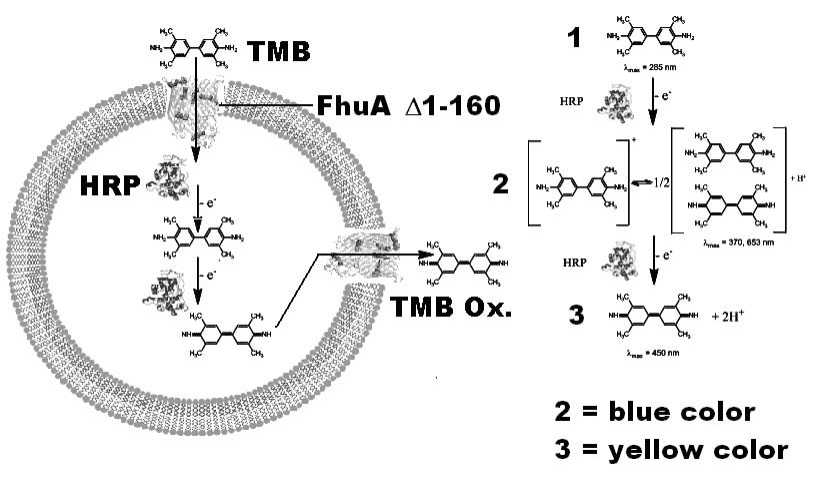
**Schematic representation of functionalized liposome system**. The FhuA Δ1-160 channel protein embedded in the liposomal lipid membrane (left panel) employed as reduction triggered gateway for the in/out diffusion of TMB and hydrogen peroxide (right panel) used in the HRP/TMB colorimetric assay.

Figure 1 shows a FhuA Δ1-160 based compound release system where FhuA Δ1-160 is embedded in a lipid membrane (left) together with the colorimetric HRP/TMB reporter used for quantifying TMB translocation (right).

### HRP based colorimetric TMB detection system

TMB as chromogen has been developed and widely used in enzyme immunoassays (EIA) employing horseradish peroxidase [13,14]. Besides, the colorimetric HRP/TMB detection system proved to be more reproducible than the previously employed calcein assay which generates a fluorescence signal upon release of self-quenching calcein from liposomes into the surrounding solution.

The HRP/TMB detection system is based on a two step consecutive oxidative reactions A→B→C (A = TMB; B and C = first and second TMB oxidation products, see Figure 1) catalyzed by HRP in presence of hydrogen peroxide. Each single step is a pseudo-second order rate reaction with a reported second order rate constant (myeloperoxidases) [14] of: k_A→B _= 3.6*10^6 ^M^-1 ^s^-1 ^and k_B→C _= 9.4*10^5 ^M^-1 ^s^-1^. The final TMB oxidation product C is unstable out of very acidic conditions [13] and the intermediate based on the first oxidation product B is used as reaction reporter, explaining the absorbance drop in time (see Additional file 1). The total amount of encapsulated HRP was not detectable though using the Soret absorption band. However kinetic data reproducibility was confirmed basing on a three data set for each measurement.

### FhuA Δ1-160 lysine positions and diffusion limited TMB translocation

**Figure 2:**
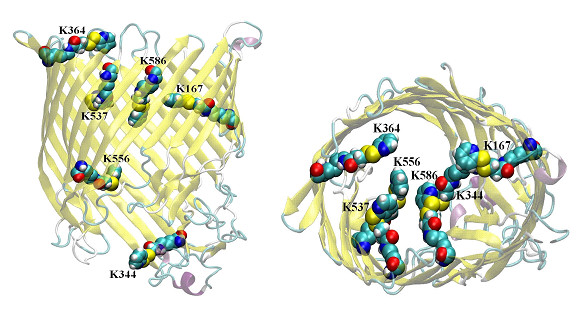
**Structural model of FhuA Δ1-160 deletion variant**. Side view (left); top view (right)) harboring the six lysine residues (K167, K344, K364, K537, K556 and K586) in the inner channel part. Lysine residues are pyridyl-labeled. FhuA Δ1-160 variant structures were energy minimized using AccelerysProgram Suite, Version 2.0 (see Additional file 1).

Figure 2 shows the six lysine residues in the FhuA Δ1-160 inner channel which upon labeling might be responsible to modulate sterically the diffusion through the channel protein. In total 12 FhuA Δ1-160 variants were generated to identify the lysine(s) which might limit TMB flux through FhuA Δ1-160 inner channel. Two subsets of six FhuA Δ1-160 variants were generated. Subset A) contains FhuA Δ1-160 variants in which one of the six lysines in the interior of FhuA Δ1-160 was substituted to alanine; subset B) contains six FhuA Δ1-160 variants in which only one lysine was not changed to alanine. Table 1 summarizes for these two subsets the TMB conversions. TMB conversions were determined by diffusion limited translocation through the FhuA Δ1-160 [12] inner channel (Additional file 1: Figure S1 and S2) using a previously reported colorimetric HRP/TMB detection system [1,13].

HRP has been entrapped in the liposome harboring FhuA Δ1-160 variants by using film hydration method coupled with extrusion. In this method, the lipid amphiphile is brought in contact with the aqueous medium containing HRP and FhuA Δ1-160 in its dry state and is subsequently hydrated to yield vesicles. After homogenization and purification of the resultant liposomes, the TMB conversion was initiated by supplementing TMB (10 μl) to the aqueous solution. Background conversions of TMB due to liposome instabilities or translocation through the membrane in absence of FhuA Δ1-160 were determined to be 13 [nM]/s (Table 1). Further control experiments were based on: liposomes harboring unlabeled FhuA Δ1-160 and the fully pyridyl-labeled FhuA Δ1-160 (starting variant). A TMB conversion of 102 [nM]/s (unlabeled FhuA Δ1-160) and 15 [nM]/s (pyridyl-labeled FhuA Δ1-160; starting variant) were reached, upon optimizing liposome preparation, and the TMB assay. The 7.9-fold higher TMB conversion in the unlabeled FhuA Δ1-160 translates an excellent detection system to monitor differences in TMB translocation through the twelve FhuA Δ1-160 variants.

### TMB conversion of the six subset A variants

The aa-position 556 has a major impact on TMB conversion: K556A substitution increases TMB conversion to 97 [nM]/s which is close to the value of the FhuA Δ1-160 unlabeled variant. A further TMB important blocking position is found by the substitution K537A increasing TMB conversion to 76 [nM]/s. In summary the following order of increased TMB conversion has been observed for subset A variants: 586 < 364 < 344 < 167 < 537 < 556.

### TMB conversion of the six subset B) variants

Subset B variants of FhuA Δ1-160 have in the inner channel only one labeled pyridyl-lysine. For pyridylated position 556, a reduction of the translocation to 16 [nM]/s was achieved. The latter proves impressively that a single labeled lysine can efficiently and independently from all other labeled lysines block TMB translocation through FhuA Δ1-160. For position 537 a cooperative effect can be observed since the subset B variant shows a significantly less pronounced TMB blocking as expected from the corresponding subset A variant. Similar to the subset A) variants the following increased TMB conversion has been observed for the subset B variants: 586 > 364 > 344 > 167 > 537 > 556.

Differences in the absolute values between the two experimental data sets can likely be attributed to pyridyl labeling efficiencies, *i.e*. inner channel sites have a lower probability to get labeled once lysines on the protein ores are labeled or when multiple sites are labeled (Additional file 1: Figure S3). Further experimental details on the TMB conversion calculations and controls (Additional file: Figure S1 and S2), CD-spectral measurements on secondary structure stability of FhuA Δ1-160 (Additional file 1: Figure S3), size measurements of liposomes (Additional file 1: Figure S4 and S5) and simulation details, can be found in the Additional file 1.

### Molecular Dynamics simulations to investigate the key modulating position 556

**Figure 3:**
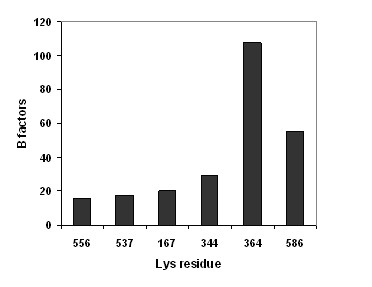
**B-factors of the Lys chains averaged on 10 ns of MD simulation**.

A working hypothesis for controlling the compound flux in the inner FhuA Δ1-160 precisely is a defined and rigid conformation of the blocking lysine residue. Lysine fluctuations of all six FhuA Δ1-160 have been directly correlated to the B factors deduced from Molecular Dynamics MD trajectories in a first simulation (see Additional file 1). The B factor analysis indicates the dynamic mobility of an atom or group of atoms. The concept is derived from the X-ray scattering/crystallography theory, alternatively known as "temperature-factor" or "Debye-Waller factor" [18]. Table 1 suggests that the amino acid position 556 is a key residue in modulating the compound flux through the inner channel. Interestingly a general trend between low B factors values of the unlabeled FhuA Δ1-160 (Figure 3) and translocation importance (experimental results; Table 1), has been found. Again the most important position 556 is in the B factor analysis the least mobile one.

In detail, FhuA Δ1-160 is a β-barrel with a cross-section of 39 Å and 46 Å on the "top" part and a reduced cross-section on the "lower" exit of the barrel, 29 Å and 19 Å. K556 is placed in a rigid β-barrel at the "lower" cross-section (Figure 2) which originally interacts with the ferrichrome peptide and TonB protein [19] for further iron translocation. Other lysines are located on the opposite site K167, K537 and K586 (placed near the top cross-section) or underneath (K344; lower cross-section). As indicated by B values the positions K556, K167 and K537 have the higher blocking effects which are in accordance to the experimental results in corresponding subset A and B variants though no simulations on the pyridyl-labeled starting variant FhuA Δ1-160, has been performed (see Additional file 1 for further discussion).

In summary, experimental results and first computational simulations indicate that the rigidity of the labeled positions play an important role in generating FhuA Δ1-160 channels with a defined and "non-fluctuating" pore size. Fluctuations in pore sizes of FhuA Δ1-160 will reduce the discriminating power to control compound fluxes and are therefore an important prerequisite for a universal compound release system that can rapidly be re-engineered to match the compound size. Following up computational simulations are required to investigate in detail the roles of the pyridyl-label, to investigate cooperative effects of labeled lysine residues and taking labeling efficiency and perturbations of protein structure after labeling into account. Further FhuA Δ1-160 engineering efforts will be based on subset B) variant K556 to further advance the control of compound fluxes through the FhuA Δ1-160 channel, especially for low molecular weight compounds.

## Conclusions

Molecular understanding of the sterically controlled diffusion in FhuA Δ1-160's inner channel is an important prerequisite to develop a universal compound release system that can rapidly be re-engineered for a "time and dose-dependent" compound release.

Six lysine residues were systematically analyzed in two subsets of engineered FhuA Δ1-160 channels. Analysis of 12 variants identified position K556 as a key substitution to sterically control compound fluxes through the inner channel of FhuA Δ1-160 embedded in liposome membrane. A first B-factor analysis based on MD simulations identified position K556 as the least fluctuating lysine among the six investigated lysines suggesting a correlation between flexibility and steric control of TMB compound translocation through the inner FhuA Δ1-160 channel. The subset B variant K556 of FhuA Δ1-160 represents therefore an excellent starting point to understand channel dynamics and to sterically control compound flux through engineered FhuA Δ1-160. Based on these results it seems promising that the reduction triggered release system can be advanced to a universal and compound independent release system which allows a size selective compound release through rationally re-engineered FhuA Δ1-160 channels.

## Methods

All chemicals used were of analytical reagent grade or higher quality, purchased from Sigma-Aldrich Chemie (Taufkirchen, Germany) and Applichem (Darmstadt, Germany) if not stated otherwise. A thermal cycler (Mastercycler gradient; Eppendorf, Hamburg, Germany) and thin-wall PCR tubes (Mμlti-ultra tubes; 0,2 ml; Carl Roth, Karlsruhe, Germany) were used in all PCRs.

### 1. Site-directed mutagenesis

Six lysines located in the FhuA Δ1-160 channel were substituted by alanine using QuikChange (developed by Stratagene; La Jolla, CA, USA) [20] derived SDM protocol generating two subsets (A & B; Table 1) of FhuA Δ1-160 variants. Table 2 lists the primers employed for SDM.

**Table 2:** Primers used for Site Directed Mutagenesis (SDM)

Sequence Name	Sequence 5' to 3'
FhuA Δ1-160 K167A Fwd	CCGCTGAAAGAAGTTCAGTTTGCGGCCGGTACTGACAGCC

FhuA Δ1-160 K167A Rev	GGCTGTCAGTACCGGCCGCAAACTGAACTTCTTTCAGCGG

FhuA Δ1-160 K344A Fwd	GGCCATTATCTGGCACGTGCGTACGTCGTTGATGATGAGAAG

FhuA Δ1-160 K344A Rev	CTTCTCATCATCAACGACGTACGCACGTGCCAGATAATGGCC

FhuA Δ1-160 K364A Fwd	GATACCCAGTTGCAGAGCGCGTTTGCCACTGGCGATATCG

FhuA Δ1-160 K364A Rev	CGATATCGCCAGTGGCAAACGCGCTCTGCAACTGGGTATC

FhuA Δ1-160 K537A Fwd	GCAGTATGAAGTCGGCGTGGCGTATGTACCGGAAGATCG

FhuA Δ1-160 K537A Rev	CGATCTTCCGGTACATACGCCACGCCGACTTCATACTGC

FhuA Δ1-160 K556A Fwd	GCCGTGTATAATCTCACTGCGACCAACAACCTGATGGCGG

FhuA Δ1-160 K556A Rev	CCGCCATCAGGTTGTTGGTCGCAGTGAGATTATACACGGC

FhuA Δ1-160 K586A Fwd	CGTAGAAATCGAAGCGGCGGCGGCGCTGTCGGCGAG

FhuA Δ1-160 K586A Rev	CTCGCCGACAGCGCCGCCGCCGCTTCGATTTCTACG

The SDM was performed by using a two-stage PCR protocol [21]: first stage: one cycle (95°C, 1 min), three cycles (95°C, 30 s; 55°C, 1 min; 68°C, 2 min) and a second stage: one cycle (95°C, 1 min), 15 cycles (95°C, 30 s; 55°C, 1 min; 68°C, 2 min) and one cycle (68°C, 25 min). In each reaction were employed template FhuA Δ1-160 (25 ng), a primer set (see Table 2; 200 nM each), dNTP mix (200 μM) and Pfu DNA polymerase (1 U) in Pfu reaction buffer (2 × 25 μl total volume, for stage one). In stage one for each primer the extension reaction is performed in a separate PCR tube and subsequently pooled for the stage 2 PCR. In Stage 2 additional Pfu DNA polymerase (0.02 U) is supplemented before starting the second PCR. For digestion of parental DNA, DpnI (10 U; 1 h, 37°C) is supplemented to the PCR mix. All 12 FhuA Δ1-160 variants were fully sequenced to assure lysine to alanine substitutions and lack of additional mutations. Amount of DNA after PCR was quantified using a NanoDrop photometer (NanoDrop Technologies, Waltham, Massachusetts, USA).

### 2. Expression, extraction and purification of FhuA variants

FhuA Δ1-160 variants were expressed, extracted and purified as previously described [1] with several modifications. pPR1-FhuA Δ1-160 plasmid is freshly transformed into the expression host *Escherichia coli *B^E ^strain BL 21 (DE3) omp8 (F^- ^*hsdS*_*B *_(r_B_^- ^m_B_^-^) *gal ompT dcm *(DE3) *Δlamb ompF::Tn5 ΔompA ΔompC*) [22] . An overnight culture (TY media, 25 ml) [12] was prepared and used to inoculate expression media (inoculate 20 ml; TY medium, 250 ml) for FhuA Δ1-160 production (1-L shaking flask; 250 rpm, 37°C, 70°C humidity//Infors HT Multitron, Bottmingen, Switzerland). When the OD_578 _reached 0.7, FhuA Δ1-160 protein expression was induced with IPTG (final concentration of 1 mM). Cells were grown (37°C) until the OD_578 _reached 2.0-2.5 and harvested (20 min, 3220 rcf, 4°C//Eppendorf 5810R; Hamburg, Germany). Cells were resuspended in lysis buffer (12 ml; pH 8.0, 20 mM Tris, 2.5 mM MgCl_2_, 0.1 mM CaCl_2_, 1 mM PMSF), cooled on ice and disrupted by passing through a high-pressure homogenizer (3×, 2000 bar//Emulsiflex-C3, Avestin Inc., Ottawa, Canada). The disrupted cell suspension was mixed with FhuA Δ1-160 extraction buffer (1 ml; pH 8.0, 20 mM phosphate buffer, 2.5 mM MgCl_2_, 0.1 mM CaCl_2_, 20% Triton X-100) and incubated (1 h, 100 rpm, 37°C//Infors HT Multitron, Bottmingen, Switzerland). The outer membrane fractions were isolated by centrifugation (45 min, 39,700 rcf, 4°C//Avanti J-20XP, Beckman Coulter, Fullerton, USA) and resuspended in pre-solubilization buffer (9 ml; pH 8.0, 20 mM phosphate buffer, 1 mM EDTA, 0.1% oPOE, 1 mM PMSF) [23]. The resuspended outer membrane fractions were subjected to a further incubation (1 h, 200 rpm, 37°C//Infors HT Multitron, Bottmingen, Switzerland) and were subsequently isolated by centrifugation (45 min, 109,760 rcf, 4°C//Beckman Optima LE-80K Ultracentrifuge, Fullerton, USA). In the final step the isolated pellet was resuspended in solubilization buffer (9 ml; pH 8.0, 20 mM phosphate, 1 mM EDTA, 3% oPOE, 1 mM PMSF) and membrane fractions were removed by centrifugation (45 min, 109,760 rcf, 4°C//Avanti J-20XP, Beckman Coulter, Fullerton, USA). The supernatant containing FhuA Δ1-160 was concentrated using ultra-filtration (20 min, 3220 rcf, RT//Eppendorf 5810R Centricon YM30; Millipore, Bedford, USA). Purity of extracted fractions was controlled by protein gel electrophoresis and comparable to previously reported values [24]. Protein concentrations were determined using the standard BCA kit (Pierce Chemical Co, Rockford, USA).

### 3. FhuA Δ1-160 labeling and nanocompartment formation

DMSO containing 3-(2-pyridyldithio) propionic acid N-hydroxysuccinimide ester (250 μl, 38 mM) was added drop-wise into FhuA Δ1-160 (750 μl, 4.3 μM) in phosphate buffer (pH 7.4, 0.2 M Na_2_HPO_4_, 0.2 M NaH_2_PO_4_, 3% oPOE) and stirred (1 h, 3000 rpm, RT//RCT basic IKAMAG, IKA-Werke GmbH, Staufen, Germany). Final concentration of DMSO and oPOE in the solution was 25% and 1.5%, respectively. The latter solution was used for formation of nanocompartments loaded with HRP (2.9 U/ml).

### 4. Liposome preparation methodology

The film hydration method coupled with the mechanical dispersion technique by filter extrusion was used [25] for liposome preparation. Conventional methods of liposome production involves three basic stages: drying of the lipid solution from organic solvents, dispersion of lipids into the aqueous media, homogenization, and purification of the resultant liposomes with subsequent analysis of the final product [26]. *E. coli *total lipid extract (Avanti Polar Lipids, Inc., Alabaster, Alabama, USA) is a chloroform extract of the respective tissue. A mixture of *E. coli *total lipid extract (500 μl, 10 mg) and methanol (1:1, v/v) were used to form a thin lipid film on round-bottom flask under reduced pressure by using a rotary evaporator (Büchi Labortechnik AG, Flawil, Switzerland). The aqueous solution containing phosphate buffer (pH 7.4, 0.2 M Na_2_HPO_4_, 0.2 M NaH_2_PO_4_), FhuA Δ1-160 (3.2 μM final concentration) and HRP (2.9 U/ml) for entrapment in the interior of the vesicles was supplemented and the thin lipid film was hydrated overnight in a 30°C water bath. Nanocompartments encapsulating HRP, harboring FhuA Δ1-160 as well as amino group labeled FhuA Δ1-160 were extruded using Avanti Lipid 1 ml syringes (Alabaster, Alabama, USA), an Avanti Lipid extrusion apparatus (Alabaster, Alabama, USA) and a Bibby Heating block (Staffordshire, UK). Three polycarbonate membranes (Millipore Corporation, Bedford, MA, USA) with pore sizes of 1 μm, 0.4 μm and 0.2 μm were used with the extrusion equipment in a sequential manner to form uniform spherically shaped nanocompartments [27]. Nanocompartments were purified by gel filtration using Sepharose 4B (Fluka, Cat. no. 84962) in phosphate buffer (pH 7.4, 0.2 M Na_2_HPO_4_, 0.2 M NaH_2_PO_4_). Average diameters of nanocompartments were routinely determined using a Zeta-Sizer (Zeta-Sizer Nano Series; Malvern, Worcestershire, UK//Additional file 1: Figure S5).

### 5. TMB assay with nanocompartments

TMB (Sigma Cat. N°: T 0440) assay was selected as a conversion reporter system. Pre-prepared TMB/H_2_O_2 _solution were used in the kinetic measurement of the TMB oxidation by the HRP [13,14]. The oxidation of TMB by the HRP/H_2_O_2 _system yields a blue and subsequently a yellow colored reaction product. Initial TMB oxidation kinetics are quantified by measuring an absorption maximum at 370 nm [14] using a microtiter plate reader (Omega Series; BMG LABTECH; Offenburg, Germany). TMB solution (10 μl) was supplemented to a 100 μl dispersion consisting of purified nanocompartments (in phosphate buffer, pH 7.4, 0.2 M Na_2_HPO_4_, 0.2 M NaH_2_PO_4_). Detailed kinetic results of the TMB assay of FhuA Δ1-160 variants are available in Additional file 1: Figure S1.

## Competing interests

The authors declare that they have no competing interests.

## Authors' contributions

AG carried out design and performed study, data analysis and drafting of the manuscript. MF and BH performed data analysis and drafting the manuscript. US carried out design, study and drafting of the manuscript. All authors read and approved the final manuscript.

## Supplementary Material

Additional file 1 **Molecular understanding of sterically controlled compound release through an engineered channel protein (FhuA)**. Additional file 1 contains a summary of kinetic data for TMB diffusion and experimental details on TMB conversion calculations and controls. Furthermore CD spectral measurements on FhuA Δ1-160 secondary structure stability, simulation details and size measurements of liposomes are presented. Click here for file

## References

[B1] Nallani M Benito S Onaca O Graff A Lindemann M Winterhalter M Meier W Schwaneberg U A nanocompartment system (synthosome) designed for biotechnological applications Journal of Biotechnology 2006 123 50 59 10.1016/j.jbiotec.2005.10.025 16364484

[B2] Nardin C Widmer J Winterhalter M Meier W Amphiphilic block copolymer nanocontainers as bioreactors The European Physical Journal E: Soft Matter and Biological Physics 2001 4 403 410 10.1007/s101890170095

[B3] Ranquin A Versees W Meier W Steyaert J Van Gelder P Therapeutic nanoreactors: combining chemistry and biology in a novel triblock copolymer drug delivery system Nano Letters 2005 5 2220 2224 10.1021/nl051523d 16277457

[B4] Meier W Nardin C Winterhalter M Reconstitution of channel proteins in (Polymerized) ABA triblock copolymer membranes Angewandte Chemie International Edition 2000 39 4599 4602 10.1002/1521-3773(20001215)39:24<4599::AID-ANIE4599>3.0.CO;2-Y 11169683

[B5] Ye JQ van den Berg B Crystal structure of the bacterial nucleoside transporter Tsx Embo Journal 2004 23 3187 3195 10.1038/sj.emboj.7600330 15272310 PMC514505

[B6] Kocer A A remote controlled valve in liposomes for triggered liposomal release Journal of Liposome Research 2007 17 219 225 10.1080/08982100701528203 18027242

[B7] Ferguson AD Hofmann E Coulton JW Diederichs K Welte W Siderophore-mediated iron transport: crystal structure of FhuA with bound lipopolysaccharide Science 1998 282 2215 2220 10.1126/science.282.5397.2215 9856937

[B8] Braun M Killmann H Braun V The beta-barrel domain of FhuA delta 5-160 is sufficient for TonB-dependent FhuA activities of *Escherichia coli* Molecular Microbiology 1999 33 1037 1049 10.1046/j.1365-2958.1999.01546.x 10476037

[B9] Braun M Killmann H Maier E Benz R Braun V Diffusion through channel derivatives of the *Escherichia coli *FhuA transport protein European Journal of Biochemistry 2002 269 4948 4959 10.1046/j.1432-1033.2002.03195.x 12383253

[B10] Koebnik R Locher KP Van Gelder P Structure and function of bacterial outer membrane proteins: barrels in a nutshell Molecular Microbiology 2000 37 239 253 10.1046/j.1365-2958.2000.01983.x 10931321

[B11] Nallani M Onaca O Gera N Hildenbrand K Hoheisel W Schwaneberg U A nanophosphor-based method for selective DNA recovery in Synthosomes Biotechnology Journal 2006 1 828 834 10.1002/biot.200600042 16927281

[B12] Onaca O Sarkar P Roccatano D Friedrich T Hauer B Grzelakowski M Güven A Fioroni M Schwaneberg U Functionalized nanocompartments (Synthosomes) with a reduction-triggered release system Angewandte Chemie International Edition 2008 47 7029 7031 10.1002/anie.200801076 18677788

[B13] Josephy PD Eling T Mason RP The horseradish peroxidase-catalyzed oxidation of 3,5,3',5'-tetramethylbenzidine. Free radical and charge-transfer complex intermediates Journal of Biological Chemistry 1982 257 3669 3675 6277943

[B14] Marquez LA Dunford HB Mechanism of the oxidation of 3,5,3',5'-tetramethylbenzidine by myeloperoxidase determined by transient- and steady-state kinetics Biochemistry 1997 36 9349 9355 10.1021/bi970595j 9235977

[B15] Szoka F Papahadjopoulos D Comparative properties and methods of preparation of lipid vesicles (liposomes) Annual Review of Biophysics and Bioengineering 1980 9 467 508 10.1146/annurev.bb.09.060180.002343 6994593

[B16] Ruysschaert T Germain M Gomes J Fournier D Sukhorukov GB Meier W Winterhalter M Liposome-based nanocapsules IEEE Transactions on Nanobioscience 2004 3 49 55 10.1109/TNB.2004.824273 15382644

[B17] Ohtsuka I Yokoyama S Penetration of bovine serum albumin into dipalmitoylphosphatidylglycerol monolayers: direct observation by atomic force microscopy Chemical & Pharmaceutical Bulletin 2005 53 42 47 10.1248/cpb.53.42 15635227

[B18] Schlessinger A Rost B Protein flexibility and rigidity predicted from sequence Proteins 2005 61 115 126 10.1002/prot.20587 16080156

[B19] Faraldo-Gomez JD Smith GR Sansom MSP Molecular dynamics simulations of the bacterial outer membrane protein FhuA: a comparative study of the ferrichrome-free and bound states Biophysical Journal 2003 85 1406 1420 10.1016/S0006-3495(03)74573-1 12944258 PMC1303317

[B20] Papworth C Braman J Wright DA QuickChange site-directed mutagenesis Strategies 1996 9 3 4

[B21] Wang WY Malcolm BA Two-stage PCR protocol allowing introduction of multiple mutations, deletions and insertions using QuikChange (TM) site-directed mutagenesis Biotechniques 1999 26 680 682 10343905 10.2144/99264st03

[B22] Prilipov A Phale PS Van Gelder P Rosenbusch JP Koebnik R Coupling site-directed mutagenesis with high-level expression: large scale production of mutant porins from *E-coli* FEMS Microbiology Letters 1998 163 65 72 10.1111/j.1574-6968.1998.tb13027.x 9631547

[B23] Locher KP Rosenbusch JP Oligomeric states and siderophore binding of the ligand-gated FhuA protein that forms channels across *Escherichia coli *outer membranes European Journal of Biochemistry 1997 247 770 775 10.1111/j.1432-1033.1997.t01-1-00770.x 9288896

[B24] Killmann H Braun V An aspartate deletion mutation defines a binding-site of the multifunctional FhuA outer-membrane receptor of *E. coli *K-12 Journal of Bacteriology 1992 174 3479 3486 1534324 10.1128/jb.174.11.3479-3486.1992 PMC206031

[B25] Gregoriadis G Preparation of liposomes 1984 1 Florida: CRC Press

[B26] Mozafari MR Liposomes: an overview of manufacturing techniques Cellular & Molecular Biology Letters 2005 10 711 719 16341279

[B27] Broz P Benito SM Saw C Burger P Heider H Pfisterer M Marsch S Meier W Hunziker P Cell targeting by a generic receptor-targeted polymer nanocontainer platform Journal of Controlled Release 2005 102 475 488 10.1016/j.jconrel.2004.10.014 15653165

