# Supplementary material for: Molecular understanding of sterically controlled compound release through an engineered channel protein (FhuA)
Source: J Nanobiotechnology. 2010 Jun 25;8:14. doi: 10.1186/1477-3155-8-14 (PMC2909997; doi:10.1186/1477-3155-8-14)
Supplement: Additional file 1 — Molecular understanding of sterically controlled compound release through an engineered channel protein (FhuA). Additional file 1 contains a summary of kinetic data for TMB diffusion and experimental details on TMB conversion calculations and controls. Furthermore CD spectral measurements on FhuA Δ1-160 secondary structure stability, simulation details and size measurements of liposomes are presented. [file 1477-3155-8-14-S1.DOC]

**Additional file 1**

Molecular understanding of sterically controlled compound release through an engineered channel protein (FhuA)

Arcan Güven^1,2^, Marco Fioroni^2^ , Bernhard Hauer^3^ and Ulrich Schwaneberg^2§^

^1^School of Engineering and Science, Jacobs University, Campus Ring 8, 28759 Bremen, Germany ^2^Lehrstuhl für Biotechnologie, RWTH Aachen University Worringerweg 1, 52074, Aachen, Germany

^3^Institut für Technische Biochemie, Universität Stuttgart, Allmandring 31, D-70569 Stuttgart, Germany


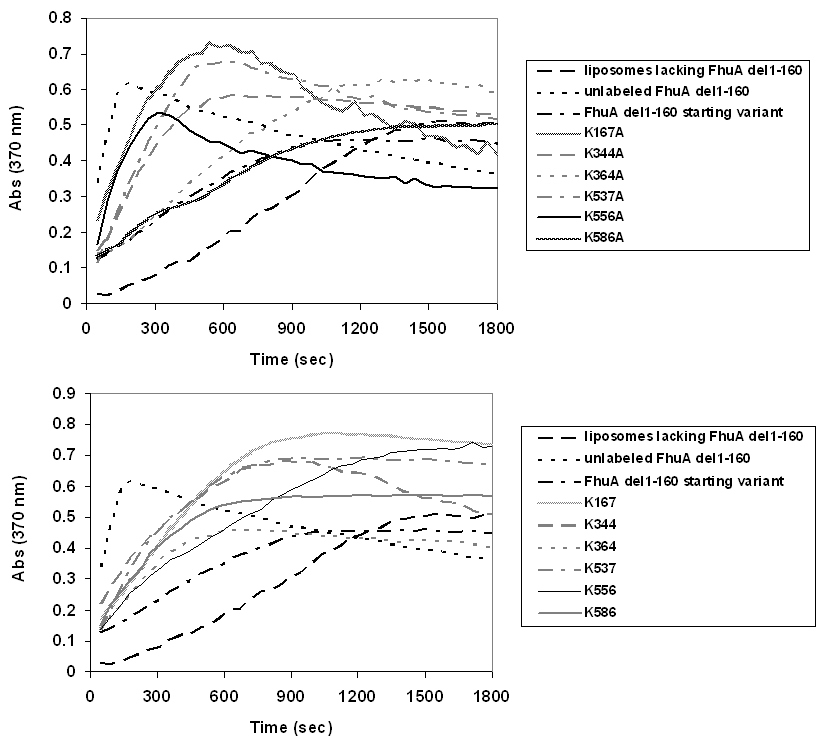


**Figure S1.** **TMB diffusion kinetics through FhuA Δ1-160 variants embedded in liposomes.** Two subsets (A & B) of FhuA Δ1-160 variants were apart from controls analyzed. FhuA Δ1-160 variants of subset A contain a single lysine to alanine substitution and of subset B contain five lysine to alanine substitutions. All FhuAΔ1-160 variants are pyridyl-labeled except two controls (liposome lacking FhuAΔ1-160 and the unlabeled FhuAΔ1-160). Upper panel: Subset A: single lysine to alanine substitution. Lower panel: Subset B: five lysine to alanine substitutions.


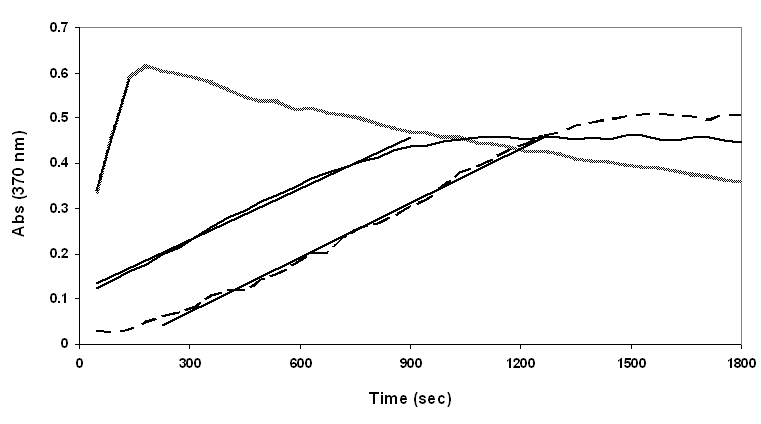


**Figure S2.** **Time derivative of the absorbance was used to calculate the TMB conversion by the Lambert-Beer law.** A linear regression using “least square” method was performed to find the best linear section in the steepest region (short-straight lines) for liposomes (dashed line), unlabeled FhuA Δ1-160 (grey line) and FhuA Δ1-160 starting variant (full line).

Lambert–Beer law, the empirical relationship that relates the absorption of light to the properties of the material through which the light is travelling was used to calculate the average TMB conversion[1]: A = εcL where A is the light absorbance, ε is the molar extinction coefficient of the substance (extinction coefficient of TMB oxidation product: 3.9x10^-4^ M^-1^ cm^-1^), c is the molar concentration of the substance and L is the distance that the light travels through the material[2] (see the manuscript, Table 1).

**Circular Dichroic Spectra of the free and pyridylated FhuA Δ1-160** Circular Dichroic (CD) spectra were registered on samples to understand the effect of the blocking chemistry on the protein secondary structure stability. Figure S3 shows the CD spectra of some of the free FhuA ∆1-160 variants and related pyridylated FhuA ∆1-160. Instrument Jasco J-815. Protein concentration: 4.3 μM. Quartz cell length: 0.05 mm.

**Figure S3.** **CD spectra of FhuA Δ1-160 (1% v/v DMSO).** Upper panel: unlabeled FhuA Δ1-160 variants; FhuA Δ1-160 (grey full-line), K167A (black dashed-line), K556A (dark grey full-line), K167 (dark grey dashed-line), FhuA Δ1-160 K556 (black full-line). Lower panel: labeled FhuA Δ1-160 variants; FhuA Δ1-160 starting variant (grey full-line), K167A (black dashed-line), FhuA K556A (dark grey full-line), K167 (grey dashed-line), FhuA Δ1-160 K556 (black full-line).

**Molecular Modeling: Crystal structure and construction of the labeled models** The crystal structure of FhuA Δ1-160 (PDB entry 1QJQ)[3] was obtained from the Protein Data Bank ([www.pdb.org](http://www.pdb.org/)). The residues 1-160, forming the channel plug, were removed from the structure. The 3-(2-pyridyldithio)propionic-acid was coupled to the 6 lysine residues of the FhuA Δ1-160 protein by the Accelerys 2.0 program version obtainable by the site: http://accelrys.com.

**Simulations Details** The simulations were carried out using the Gromacs 3.3.3 program package[4] using the ffG45a3 force field[5]. The FhuA Δ1-160 protein was neutralized with 14 Na^+^ counterions placed at the most negative positions close to the protein than surrounded by 114 detergent molecules, n-octyl-2-hydroxyethyl sulfoxide (n=8). Topologies were built by the PRODRG program [6].

A dodecahedron based box of 22473 SPC water molecules was used to solvate the protein respecting the minimum image rule to avoid spurious self-interactions.

The minimizations were performed using the steepest descent algorithm. Equilibrations were performed maintaining all residues restrained by a force constants gradually reduced from 500 to 0 kcal/mol in 100000 steps while the rest of the system was allowed to relax for a total of 200 ps at a T=50 K. The system was then heated from 50 to 300 K in 100 ps. The production runs of 50 ns were carried out at 300 K in the NPT ensemble at constant-pressure by the use of the Parrinello-Rahmann barostat with P=1 bar and τ_P_=2.5 ps and at constant temperature by the use of the Nose-Hoover with τ_T_= 1.0 ps. Long range electrostatic interactions were treated by the Particle Mesh Ewald (PME) with cut-offs 0.9 nm in parallel with the VdW interactions with a cut-off of 0.9 nm The center of mass velocity was periodically removed during the production dynamics. Trajectories were analyzed using the Gromacs[5] tools.

**Domain analysis and decomposition in FhuA Δ1-160** The main variables influencing the ability of subset A and subset B variants to influence TMB translocation have been correlated to the secondary structure, domain flexibility and distance from the ores (see Figure 2). Secondary structure flexibility is directly correlated to low and high amplitudes of backbone fluctuations existing in the FhuA Δ1-160 β-barrel and in random coils in cork interacting regions, while domain flexibility refers to fluctuations of parts of FhuA. In fact a domain may include different secondary structures or sub-domains though stable, move in precise directions, giving an anisotropic character to the protein fluctuations itself. The distance from an ore defines the ability of the label compound to mainly fluctuate near the channel entrance. Shorter label distances to a channel ore imply better closing efficiencies.

For completeness, based on the above explanation first neighbor aminoacids to K 556 are not expected to behave differently reporting comparable fluctuations. For example T 557 or N 558 has similar B factors values (22 and 23 respectively) compared to K 556 (18). In fact they are still in the same protein domain of K 556 and near to one of the protein ores. However, due to the Lys numbers (n=19) and locations (as explained in page 4, first 6 lines in the main text and in Onaca et. al., 2008, Ref # 12 in the main text) compared, for example, to Ser (n=40) or CysH (n=0), the coupling chemistry was developed considering the amino group labeling. As a consequence the MD B-factor analysis has been reported only for the Lys groups because of the possible comparison/correlation to the experimental data.

**DLS Data Analysis and Results** Dynamic Light Scattering (DLS) data for liposomes encapsulating HRP, liposomes encapsulating HRP and accommodating open FhuA Δ1-160 and liposomes encapsulating HRP and accommodating close FhuA Δ1-160 were obtained (Figure S4). Furthermore although a detailed study on stability of liposomes was not performed the aggregation of liposomes was observed after three weeks (Figure S5).


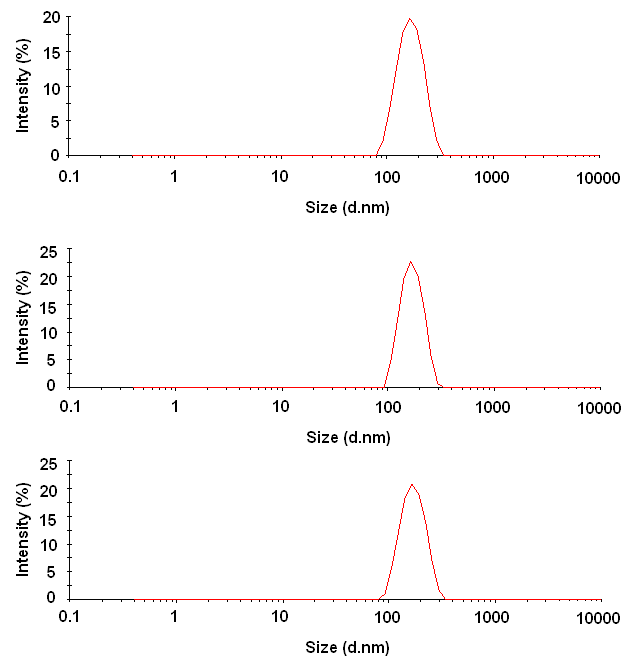


**Figure S4.** **Average liposome size distribution.** Upper panel: liposomes encapsulating HRP (170.7 nm; 98 %). Middle panel: liposomes encapsulating HRP and accommodating completely open FhuA Δ1-160 channel (161.5 nm; 97.2 %). Lower panel: liposomes encapsulating HRP and accommodating blocked FhuA Δ1-160 channel (172.1 nm; 96.3 %).


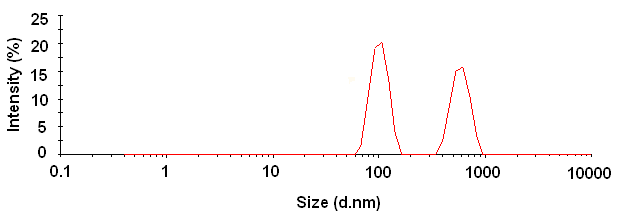


**Figure S5.** **Average size distribution of liposomes.** First peak: 102.2 nm (54.9 %) and second peak: 588.4 nm (45.1 %)) of liposomes encapsulating HRP after three weeks.

**References**

1. Gouvêa A, Pereira AT, Pimentel AC, Prazeres DMF, Chu V, Conde JP: **Colorimetric detection of molecular recognition reactions with an enzyme biolabel using a thin-film amorphous silicon photodiode on a glass substrate.** *Sensors and Actuators B: Chemical* 2008, **135:**102-107.

2. Josephy PD, Eling T, Mason RP: **The horseradish peroxidase-catalyzed oxidation of 3,5,3',5'- tetramethylbenzidine. Free radical and charge-transfer complex intermediates.** *J Biol Chem* 1982, **257:**3669-3675.

3. Andrew D. Ferguson JWCKDWWVBH-PF: **Crystal structure of the antibiotic albomycin in complex with the outer membrane transporter FhuA.** *Protein Science* 2000, **9:**956-963.

4. Kutzner C, Spoel Dvd, Fechner M, Lindahl E, Schmitt UW, Groot BLd, Grubmuller H: **Speeding up parallel GROMACS on high-latency networks.** *J Comp Chem* 2007, **28:**2075-2084.

5. Spoel Dvd, Lindahl E, Hess B, Groenhof G, Mark AE, Berendsen HJC: **GROMACS: Fast, Flexible and Free.** *J Comp Chem* 2005, **26:**1701-1718.

6. Schuettelkopf AW, Aalten DMFv: **PRODRG - a tool for high-throughput crystallography of protein-ligand complexes.** *Acta Crystallographica D* 2004, **60:**1355—1363.
